# Marketing and Psychology: An Interdisciplinary Partnership

**DOI:** 10.1093/geroni/igab046.401

**Published:** 2021-12-17

**Authors:** Melinda Heinz, Summer Zwanziger Elsinger

**Affiliations:** Upper Iowa University, Fayette, Iowa, United States

## Abstract

Students enrolled in Psychology of Aging and Consumer Behavior combined efforts during an 8-week course to create marketing plans focused on proposing a product or service targeting older adults. The goal of the project was to 1) infuse aging content into the curriculum 2) increase awareness of older adult consumers and 3) decrease aging stereotypes. Student teams were engaged in this project one day each week over 8 weeks. Instructors created weekly tasks to break down components of the project and each student group was required to turn in evidence of their completed task. During the 2020-2021 academic year, participants used Microsoft Teams and recorded their tasks for instructors to grade. A rubric was used to facilitate grading of weekly team tasks and similar weights/points were used in both classes to create similar levels of student “buy in.” Suggested implementation tips for both face-to-face and online modalities will be discussed.

